# 8-Hydroxybriaranes from Octocoral *Briareum stechei* (Briareidae) (Kükenthal, 1908)

**DOI:** 10.3390/md19030136

**Published:** 2021-02-28

**Authors:** Thanh-Hao Huynh, Su-Ying Chien, Junichi Tanaka, Zhi-Hong Wen, Yang-Chang Wu, Tung-Ying Wu, Ping-Jyun Sung

**Affiliations:** 1 Department of Marine Biotechnology and Resources, National Sun Yat-sen University, Kaohsiung 804201, Taiwan; d095020008@student.nsysu.edu.tw (T.-H.H.); wzh@mail.nsysu.edu.tw (Z.-H.W.); 2 National Museum of Marine Biology and Aquarium, Pingtung 944401, Taiwan; 3 Instrumentation Center, National Taiwan University, Taipei 106319, Taiwan; suyingchien@ntu.edu.tw; 4 Department of Chemistry, Biology and Marine Science, University of the Ryukyus, Nishihara, Okinawa 9030213, Japan; jtanaka@sci.u-ryukyu.ac.jp; 5 Institute of BioPharmaceutical Sciences, National Sun Yat-sen University, Kaohsiung 804201, Taiwan; 6 Graduate Institute of Integrated Medicine, College of Chinese Medicine, China Medical University, Taichung 404333, Taiwan; yachwu@mail.cmu.edu.tw; 7 Chinese Medicine Research and Development Center, China Medical University Hospital, Taichung 404394, Taiwan; 8 Department of Biological Science & Technology, Meiho University, Pingtung 912009, Taiwan; 9 Department of Food Science and Nutrition, Meiho University, Pingtung 912009, Taiwan; 10 Graduate Institute of Marine Biology, National Dong Hwa University, Pingtung 944401, Taiwan; 11 Graduate Institute of Natural Products, Kaohsiung Medical University, Kaohsiung 807378, Taiwan

**Keywords:** *Briareum stechei*, briarane, briastecholide, solenolide, anti-inflammation, iNOS

## Abstract

Chemical investigation of the octocoral *Briareum stechei*, collected in the Ie Island, Okinawa, Japan, resulted in the isolation of a new briarane-type diterpenoid, briastecholide A (**1**), as well as the previously reported metabolites, solenolide C (**2**) and briarenolide S (**3**). The structures of briaranes **1**–**3** were characterized through spectroscopic analysis, and the absolute configuration of **2** was corroborated by a single-crystal X-ray diffraction analysis. Briarane **3** exhibited bioactivity against the protein expression of inducible nitric oxide synthase (iNOS).

## 1. Introduction

**Figure 1:**
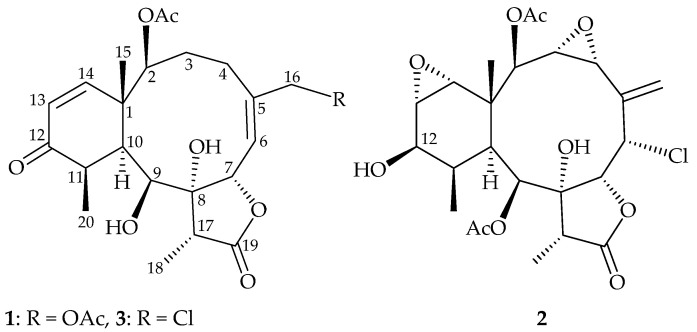
Structures of briastecholide A (**1**), solenolide C (**2**) and briarenolide S (**3**).

Octocorals of the genus *Briareum* (family Briareidae), flourishing in the tropical and subtropical Indo-Pacific Ocean, are proven to be the most important sources to yield the 3,8-cyclized cembranoids (briarane), and compounds of this type were found to possess complex structures and extensive bioactivities, especially in anti-inflammatory activity [1,2]. *Briareum stechei* (Kükenthal, 1908) [3], one of the widely studied species of octocoral, is considered a rich source of briarane-type compound. In continuation of our interest in the comparative chemistry of *Briareum* species collected at diverse geographical locations, we obtained a specimen of *B. stechei* from Ie Island, Okinawa, Japan [3,4]. We report herein the isolation, structure determination and antiinflammatory activity of one new (briastecholide A (**1**)), one revised (solenolide C (**2**)) [5,6] and one known (briarenolide S (**3**)) [7] briarane (Figure 1). 

## 2. Results and Discussion

Freshly collected *B. stechei* was frozen and subsequently freeze-dried, powdered and extracted with a mixture of methanol/dichloromethane (MeOH/CH_2_Cl_2_) at a 1:1 ratio to produce an extract that was separated by ethyl acetate (EtOAc)–water partitioning. The EtOAc layer was collected and loaded onto a column chromatograph with silica gel, and separated using high performance liquid chromatography (HPLC), yielding briaranes **1**–**3**.

**Figure 2:**
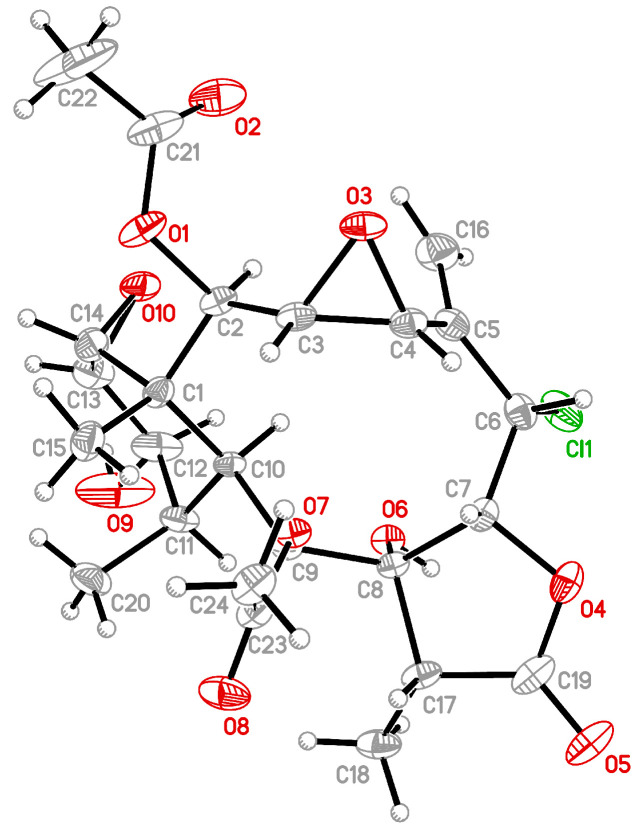
Crystal structure and absolute configuration of solenolide C (**2**) by X-ray diffraction.

Solenolide C (**2**) was first isolated from octocoral *Solenopodium* sp. [5], and the stereochemistry of C-12 in this compound was revised in a later study by NMR data analysis [6]. The structure of **2** was determined directly in this study for the first time by a single-crystal X-ray analysis, and the Oak Ridge Thermal Ellipsoid Plot (ORTEP) diagram (Figure 2) showed that the absolute configurations of stereogenic carbons of **2** are 1*R*,2*R*,3*R*,4*R*,6*S*,7*R*,8*R*,9*S*,10*S*,11*R*,12*R*,13*S*,14*R* and 17*R*. Analyzing the X-ray structure of **2** confirmed the β-orientation of the 12-hydroxy group, rather than an α-orientation, as stated in a previous study [5] (Supplementary Materials, Figures S12–S16).

Briastecholide A (**1**) was obtained as an amorphous powder. The positive mode high resolution electrospray ionization mass spectrum [(+)-HRESIMS] showed a peak at *m/z* 487.19356, suggesting a molecular formula C_24_H_32_O_9_ (calcd. for C_24_H_32_O_9_ + Na, 487.19385) (nine degrees of unsaturation). The IR spectrum indicated the presence of hydroxy (ν_max_ 3462 cm^−1^), γ-lactone (ν_max_ 1771 cm^−1^), ester carbonyl (ν_max_ 1740 cm^−1^) and α,β-unsaturated ketonic (ν_max_ 1675 cm^−1^) groups. The ^13^C NMR spectrum of **1** (Table 1) showed signals of 24 carbons. The multiplicity of carbon signals was determined from distortionless enhancement by polarization transfer (DEPT) and heteronuclear single quantum coherence (HSQC) spectra: five methyls, three methylenes (one bearing a heteroatom), nine methines (three bearing a heteroatom and three olefins) and seven non-protonated carbons (four carbonyls, one olefin and one bearing a heteroatom). From ^13^C and ^1^H NMR spectra (Table 1), **1** was found to possess a γ-lactone (δ_C_ 177.6, C-19), two acetoxy (δ_H_ 2.29, 2.05, each 3H × s; δ_C_ 20.8, 2 × acetate methyls; δ_C_ 170.6, 167.7, 2 × acetate carbonyls), an α,β-unsaturated ketonic (δ_H_ 6.29, 1H, d, *J* = 10.4 Hz, H-14; 5.98, 1H, d, *J* = 10.4 Hz, H-13; δ_C_ 202.3, ketonic carbonyl, C-12; 154.0, CH-14; 126.3, CH-13) and an trisubstituted olefin (δ_H_ 5.73, 1H, d, *J* = 9.6 Hz, H-6; δ_C_ 140.5, C-5; 124.4, CH-6) moiety. Six double bonds accounted for six unsaturated degrees. The remaining three degrees of unsaturation defined **1** as a tricyclic molecule.

**Figure 3:**
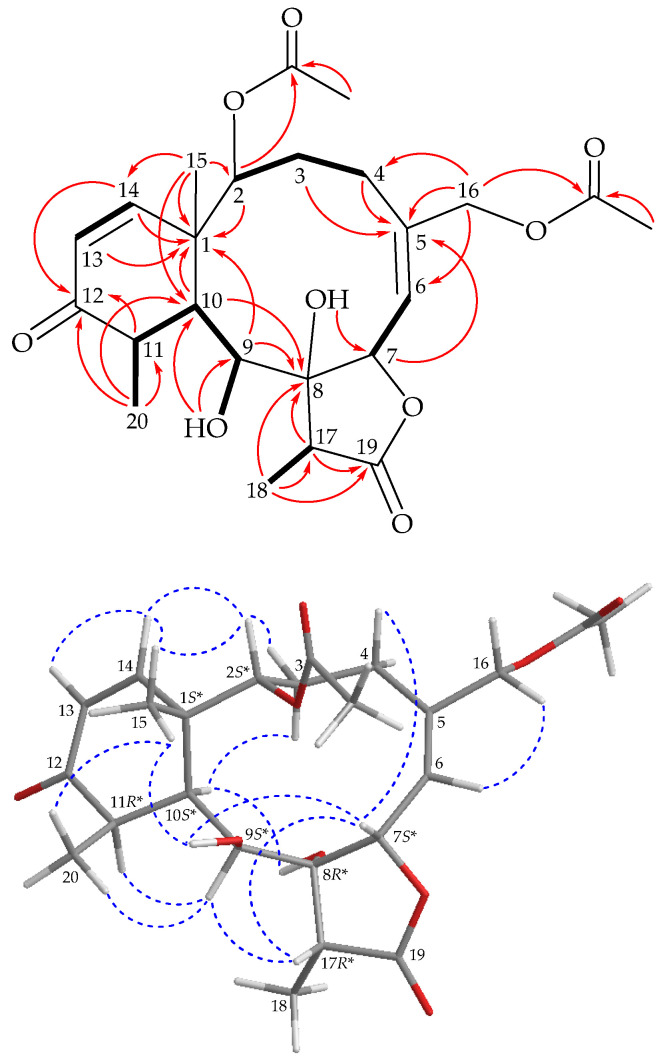
Key COSY (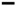), HMBC (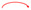) and protons with NOESY (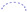) correlations of **1**.

The H-2/H_2_-3/H_2_-4, H-6/H-7, H-9/H-10/H-11/H_3_-20, H-13/H-14 and H-17/H_3_-18 spin systems, measured in the ^1^H–^1^H correlation spectroscopy (COSY) (Figure 3), were fit to the regiochemistry of vicinal proton couplings in **1**. The tricyclic network was established by heteronuclear multiple bond coherence (HMBC) experiments, especially by ^2^*J*- and ^3^*J*-^1^H–^13^C long-range correlations between protons and non-protonated carbons, such as H-2, H-9, H-10, H-13, H-14, H_3_-15/C-1; H_2_-3, H_2_-4, H-7, H_2_-16/C-5; H-9, H-10, H-17, H_3_-18/C-8; H-11, H-14, H_3_-20/C-12; and H-17, H_3_-18/C-19, thus permitting the elucidation of the carbon skeleton of **1** (Figure 3). Methyl groups Me-15, Me-18 and Me-20 at C-1, C-17 and C-11 were confirmed by the HMBC correlations between H_3_-15/C-1, C-2, C-10, C-14; H_3_-18/C-8, C-17 and C-19 and H_3_-20/C-10, C-11 and C-12, respectively. Acetate esters at C-2 and C-16 were established from the correlations between oxymethine proton H-2 (δ_H_ 4.91) and oxymethylene protons H_2_-16 (δ_H_ 4.46) and acetate carbonyls resonating at δ_C_ 167.7 and 170.6, respectively, observed in the HMBC spectrum of **1**. The hydroxy proton signal at δ_H_ 4.54 (1H, d, *J* = 9.2 Hz, OH-9) was revealed by its ^1^H–^1^H COSY and HMBC correlations to H-9 (δ_H_ 3.75, 1H, dd, *J* = 9.2, 7.2 Hz) and δ_C_ 69.3 (CH-9), respectively, indicating its attachment to C-9. Eight of the nine oxygen atoms in the molecular formula of **1** could be accounted for by the presence of a γ-lactone, two esters, an α,β-unsaturated ketonic group and a hydroxy group. Thus, the remaining oxygen atom had to be positioned at C-8, an oxygen-bearing, non-protonated carbon, as a hydroxy group, as indicated by its ^13^C NMR chemical shifts resonating at δ_C_ 84.8. Based on the above findings, the planar structure of **1** was established.

The stereochemical evaluation of **1** was established using a nuclear Overhauser effect spectroscopy (NOESY) experiment (Figure 3). In naturally occurring briaranes, proton H-10 and Me-15 at C-1 are α- and β-oriented, respectively. In the NOESY experiment, H_3_-15 correlated with OH-9 and H_3_-20, while OH-9 correlated with H-7, indicating that these protons were situated on the same face, and were assigned as β protons. A NOESY correlation observed between H-7 and H-17 reflected that H-17 and 8-hydroxy groups were β- and α-oriented in the γ-lactone moiety, respectively, by modeling analysis. The *cis* geometry of the C-13/14 double bond was indicated by a 10.4 Hz coupling constant between H-13 (δ_H_ 5.98) and H-14 (δ_H_ 6.29), and further confirmed by a NOESY correlation between these two olefin protons. Furthermore, H-2 showed NOESY correlations with H-14 and H_3_-15, demonstrating the S*-configuration of the stereogenic center C-2. H-6 exhibited a cross peak with H_2_-16, but not with H-7, and a large coupling constant (*J* = 9.6 Hz) was detected between H-6 and H-7, indicating that the dihedral angle between H-6 and H-7 was approximately 180°, and the *E*-geometry of C-5/6 double bond was elucidated. It was found that the NMR data of **1** were similar to those of a known briarane, briarenolide S (**3**) [7] (Figure 1), which was also obtained in this study, except that the signals corresponding to the 16-chlorine group in **3** were replaced by those of an acetoxy group in **1**. Therefore, briastecholide A (**1**) was assigned as having a structure with the same relative stereochemistry as briarenolide S (**3**) because of the stereogenic carbons that **1** has in common with **3**, and the configurations of the stereogenic centers of **1** were elucidated as (1S*,2S*,7S*,8R*,9S*,10S*,11R* and 17R*) (Supplementary Materials, Figures S1–S11).

Briarane **3** was obtained as an amorphous powder. The (+)-ESIMS mass showed a pair of peaks at *m/z* 463/465 ([M + Na]^+^/[M + 2 + Na]^+^) (3:1), with a relative intensity suggestive of a chlorine atom, and was found to have the molecular formula C_22_H_29_ClO_7_ by analysis of ^13^C and ^1^H NMR data. The result revealed that this compound had eight degrees of unsaturation. Strong bands at 3459, 1753 and 1735 cm^−1^ in the IR spectrum indicated the presence of hydroxy, γ-lactone and ester groups. The ^13^C NMR and DEPT spectra revealed that **3** had 22 carbons, including four methyls, three sp^3^ methylenes, six sp^3^ methines, three sp^2^ methines, two sp^3^ non-protonated carbons and four sp^2^ non- protonated carbons. Therefore, **3** was identified as having three rings. It was found that the spectroscopic data of **3** were identical to those of a known briarane, briarenolide S [7], and these two compounds possessed negative optical rotation values, suggesting that compound **3** is briarenolide S (Supplementary Materials, Figures S17–S21).

The effects of briaranes **1**–**3** on the release of inducible nitric oxide synthase (iNOS) and cyclooxygenase-2 (COX-2) from lipopolysaccharide(LPS)-stimulated RAW 264.7 macrophage cells were assessed (Table 2). Briarane **3** at 10 μM suppressed the release of iNOS to 66.5 ± 3.4%, as compared to the results of the cells stimulated with LPS only, and by comparison of the activities of **1** with those of **3**, it was found that **3** was more active in terms of reducing the expression of iNOS, indicating that the activity of **1** and **3** is largely dependent on the functional group at C-16.

## 3. Materials and Methods

### 3.1. General Experimental Procedures

For IR spectra, a Nicolet iS5 FT-IR spectrophotometer (Thermo Fisher Scientific, Waltham, MA, USA) was used. Optical rotation values were measured using a Jasco P-1010 digital polarimeter (Jasco, Tokyo, Japan). NMR spectra were measured with a Jeol ECZ 400 MHz spectrometer (Jeol, Tokyo, Japan). ESIMS and HRESIMS analyses were conducted using the Bruker 7 Tesla solariX FTMS system (Bruker, Bremen, Germany). Column chromatography was carried out with silica gel (230–400 mesh, Merck, Darmstadt, Germany). Thin layer chromatography (TLC) was performed on plates precoated with Kieselgel 60 F_254_ (0.25 mm, Merck), then sprayed with 10% H_2_SO_4_ solution followed by heating to visualize the spots. Normal-phase high performance liquid chromatography (NP-HPLC) was performed using a system comprising a pump (L-7110, Hitachi, Tokyo, Japan), an injection port (Rheodyne 7725i; Rohnert Park, CA, USA) and a semi-preparative normal-phase column (YMC-Pack SIL, 250 × 20 mm, 5 μm; Sigma-Aldrich, St. Louis, MO, USA). Reverse-phase HPLC (RP-HPLC) was performed using a system comprising a Hitachi L-2130 pump, a Hitachi L-2455 photodiode array detector, a Rheodyne 7725i injection port and a semi-preparative reverse-phase column (Luna, 5 μm, C18(2) 100Å, AXIA, 250 × 21.2 mm; Phenomenex, Torrance, CA, USA).

### 3.2. Animal Material

Specimens of *Briareum stechei* used for this study were collected in the Ie Island, Okinawa, Japan (N26.44.21.8, E127.48.33.8) in 2019. The coral specimen was identified as *Briareum stechei* (Kükenthal, 1908) based on its morphology and micrographs of the coral sclerites [3,4]. The samples were then stored at −20 °C until extraction.

### 3.3. Extraction and Isolation

Freeze-dried and sliced bodies (wet/dry weight = 618/305 g) of the coral specimen were extracted at room temperature with MeOH/CH_2_Cl_2_ (1:1). The extract was concentrated under reduced pressure to produce 42.7 g crude extract, which was partitioned between EtOAc and H_2_O. The EtOAc layer (15.1 g) was applied to column chromatography on silica gel and eluted with gradients of hexanes/EtOAc. By TLC monitoring and by using sulfuric acid spray reagent, 11 fractions A−K were obtained. Fraction F was separated by NP-HPLC using a mixture of *n*-hexane/EtOAc (3:2) to yield fractions F1−F10. Fraction F8 was re-purified by RP-HPLC using a mixture of MeOH/H_2_O (60:40) (flow rate = 5.0 mL/min) to afford **3** (1.5 mg). Purification of fraction G was performed by NP-HPLC using a mixture of *n*-hexane/acetone (3:1) to yield fractions G1−G10. Fraction G7 and G8 were re-purified by RP-HPLC using a mixture of MeOH/H_2_O (60:40 for G7, 55:45 for G8; flow rate = 5 mL/min) to afford **1** (1.2 mg) and **2** (6.0 mg), respectively.

Briastecholide A (**1**): Amorphous powder; [α]${}_{D}^{22}$ −30 (*c* 0.1, CHCl_3_); IR (KBr) ν_max_ 3462, 1771, 1740, 1675 cm^−1^; ^1^H (400 MHz, CDCl_3_) and ^13^C (100 MHz, CDCl_3_) NMR data, see Table 1; ESIMS: *m/z* 487 [M + Na]^+^; HRESIMS: *m/z* 487.19356 (calcd. for C_24_H_32_O_9_ + Na, 487.19385).

Solenolide C (**2**): Colorless prisms; [α]${}_{D}^{20}$ −40 (*c* 0.3, MeOH) ([5] [α]${}_{D}^{20}$ −25 (*c* 0.76, MeOH)); IR (ATR) ν_max_ 3538, 1764, 1741 cm^−1^; the ^1^H and ^13^C NMR data of **2** are in full agreement with those reported previously [5]; ESIMS: *m/z* 537 [M + Na]^+^, 539 [M + 2 + Na]^+^.

Briarenolide S (**3**): Amorphous powder; [α]${}_{D}^{20}$ −8 (*c* 0.1, CHCl_3_) ([7] [α]${}_{D}^{20}$ −4 (*c* 0.2, CHCl_3_)); IR (ATR) ν_max_ 3459, 1753, 1735 cm^−1^; the ^1^H and ^13^C NMR data of **3** are in full agreement with those reported previously [7]; ESIMS: *m/z* 463 [M + Na]^+^, 465 [M + 2 + Na]^+^.

### 3.4. Single-Crystal X-ray Crystallography of Solenolide C (**2**)

The structures of **2** were determined by single-crystal X-ray diffraction on two dual source equipped Bruker D8 Venture diffractometers. Solenolide C (**2**) was crystallized from methanol as colorless prisms in the monoclinic crystal system in the *P*2_1_ (#4) space group—the crystal (0.211 × 0.174 × 0.163 mm^3^) with *a* = 10.07780(10) Å, *b* = 14.4322(2) Å, *c* = 19.5756(2) Å, *V* = 2761.45(6) Å^3^, *Z* = 2, *D*_calcd_ = 1.277 Mg/m^3^, *λ* (Mo Kα) = 0.71073 Å. All 33,970 reflections were collected. The structure was solved by direct methods and refined by a full-matrix least-squares procedure. The refined structural model converged to a final *R*1 = 0.0556; w*R*2 = 0.1579 for 16054 observed reflections [*I* > 2*σ*(*I*)] and 671 variable parameters. Absolute configuration could be determined reliably for compound **2** with Flack’s parameter of 0.02(2). Full crystallographic data can be retrieved from the CIF (crystallographic information files) file CCDC (Cambridge Crystallographic Data Centre) 2056551 containing the supplementary crystallographic data for this paper [8]. These data can be obtained free of charge from the CCDC via www.ccdc.cam.ac.uk/structures/ (accessed on 16 January 2021).

### 3.5. In Vitro Inflammatory Assay

Pro-inflammatory proteins inducible nitric oxide synthase (iNOS) and cyclooxygenase-2 (COX-2) in macrophages were induced by incubating them for 16 h in a medium containing LPS (0.01 μg/mL) without compounds. For the anti-inflammatory activity assay, compounds or positive control (dexamethasone) were added to the cells 5 min before the lipopolysaccharides (LPS) administrate. After exposure to the compounds or dexamethasone, the macrophages were washed with ice-cold phosphate-buffered saline (PBS), lysed in ice-cold lysis buffer (50 mM Tris, pH 7.5, 150 mM NaCl, 1% Triton X-100, 100 μg/mL phenylmethylsulfonyl fluoride and 1 μg/mL aprotinin) and centrifuged at 20,000× *g* for 30 min at 4 °C. The supernatants were decanted and reserved for Western blotting. Protein concentrations were measured using a protein assay kit (Bio-Rad, Hercules, CA, USA). The method of Western blotting was similar to that in our previous study [8]. Anti-β-actin antibody was obtained from Sigma Chemical (St. Louis, MO, USA). Anti-iNOS and anti-COX-2 antibodies were purchased from Cayman Chemical Company (Ann Arbor, MI, USA). Horseradish peroxidase-conjugated secondary antibodies were obtained from Jackson ImmunoResearch Laboratories (West Grove, PA, USA). The images of Western blotting were obtained using the UVP BioChemi Imaging System (UVP, Upland, CA, USA). Relative densitometric quantification of the Western blotting band was performed using LabWorks 4.0 software (UVP LLC, Upland, CA, USA). The intensity of the LPS only group was set at 100%. The β-actin was used as the loading/internal control.

## 4. Conclusions

A new briarane, briastecholide A (**1**), and two known briarane analogues, solenolide C (**2**) and briarenolide S (**3**), were identified from octocoral *B. stechei*, collected in Ie Island, Okinawa, Japan, where the Kuroshio current and South China Sea surface current converge to provide high biodiversity. Briaranes **1** and **3** were isolated along with **2** from the same target organism, *B. stechei*, and the absolute configuration of **2** was determined by X-ray diffraction analysis. Therefore, it is biogenetically reasonable to conclude that **1** and **3** have the same absolute configuration as **2**, and the configurations of the stereogenic centers of **1** were elucidated as 1*S*,2*S*,7*S*,8*R*,9*S*,10*S*,11*R* and 17*R*. Briarane **3** displayed reducing effects on the production of iNOS at a concentration of 10 μM.

## Figures and Tables

**Table 1:** ^1^H and ^13^C NMR data for briarane **1**.

Position.	δ_H_^a^ (*J* in Hz)	δ_C_^b^, Mult.
1		45.1, C ^c^
2	4.91 d (9.6)	81.3, CH
3α/β	1.66 dd (15.6, 7.2); 2.35 m	24.3, CH_2_
4α/β	1.96 m; 2.43 m	20.8, CH_2_
5		140.5, C
6	5.73 d (9.6)	124.4, CH
7	5.27 d (9.6)	76.1, CH
8		84.8, C
9	3.75 dd (9.2, 7.2)	69.3, CH
10	2.95 dd (7.2, 4.0)	38.7, CH
11	2.98 qd (7.2, 4.0)	44.4, CH
12		202.3, C
13	5.98 d (10.4)	126.3, CH
14	6.29 d (10.4)	154.0, CH
15	1.44 s	20.1, CH_3_
16	4.46 br s	67.1, CH_2_
17	3.43 q (7.2)	41.9, CH
18	1.21 d (7.2)	6.4, CH_3_
19		177.6, C
20	1.25 d (7.2)	14.5, CH_3_
OH-8	3.24 s	
OH-9	4.54 d (9.2)	
OAc-2		167.7, C
	2.29 s	20.8, CH_3_
OAc-16		170.6, C
	2.05 s	20.8, CH_3_

^a^ 400 MHz, CDCl_3_, ^b^ 100 MHz, CDCl_3_, ^c^ multiplicity deduced by ^13^C and DEPT spectra.

**Table 2:** Suppression effects of cembranoids **1**–**3** on iNOS and COX-2 protein/enzyme expressions in LPS-induced macrophages.

Compound/Treatment	iNOS	COX-2	β-Actin	*n*
(10 µM)	Production Level			
Control	2.2 ± 0.9	1.0 ± 0.1	106.1 ± 4.2	4
Vehicle	100.0 ± 4.3	100.0 ± 2.6	100.0 ± 0.7	4
**1**	88.3 ± 0.3	101.8 ± 3.1	100.4 ± 5.3	4
**2**	88.9 ± 2.9	94.7 ±3.2	101.1 ± 4.4	4
**3**	66.5 ± 3.4	112.3 ±5.8	99.3 ± 4.4	4
Dexamethasone	54.5 ± 3.6	17.7 ±1.8	103.1 ± 2.5	4

Values of cells treated with LPS alone were set to 100% as the reference for normalization. Dexamethasone at 10 µM was used as a positive reference to treat cells. Experimental results are shown as the mean ± S.E.M.

## Data Availability

The data presented in this study are available on request from the corresponding author.

## Supplementary Materials

Supplementary Materials are available online at https://www.mdpi.com/1660-3397/19/3/136/s1: ESIMS, IR, 1D (^1^H and ^13^C) and DEPT NMR spectra of briaranes **1**–**3**; HRESIMS and 2D (HSQC, HMBC, COSY and NOESY) NMR spectra of **1**.
