# Supplementary material for: 8-Hydroxybriaranes from Octocoral Briareum stechei (Briareidae) (Kükenthal, 1908)
Source: Mar Drugs. 2021 Feb 28;19(3):136. doi: 10.3390/md19030136 (PMC7998228; doi:10.3390/md19030136)
Supplement: Supplementary file 1 [file marinedrugs-19-00136-s001.pdf]

Supplementary materials

8-Hydroxybriaranes from Octocoral *Briareum stechei* (Briareidae) (Kükenthal, 1908)

Thanh-Hao Huynh [1,2], Su-Ying Chien [3], Junichi Tanaka [4], Zhi-Hong Wen [1,5], Yang-Chang Wu [6,7], Tung-Ying Wu [8,9,*] and Ping-Jyun Sung [1,2,7,10,11,*]

S1. ESIMS spectrum of compound **1** 2

S2. HRESIMS spectrum of compound **1** 3

S3. IR spectrum of compound **1** 3

S4. ^{1}H NMR spectrum (400 MHz) of compound **1** in $CDCl_3$ 4

S5. ^{13}C NMR spectrum (100 MHz) of compound **1** in $CDCl_3$ 4

S6. DEPT spectrum of compound **1** in $CDCl_3$ 5

S7. HSQC spectrum of compound **1** in $CDCl_3$ 5

S8. HMBC spectrum of compound **1** in $CDCl_3$ 6

S9. ^{1}H-^{1}H COSY spectrum of compound **1** in $CDCl_3$ 6

S10. NOESY spectrum of compound **1** in $CDCl_3$ 7

S11. NOESY spectrum of compound **1** in CD_3OD 7

S12. ESIMS spectrum of compound **2** 8

S13. IR spectrum of compound **2** 9

S14. ^{1}H NMR spectrum (400 MHz) of compound **2** in CD_3COCD_3 9

S15. ^{13}C NMR spectrum (100 MHz) of compound **2** in CD_3OD 10

S16. DEPT spectrum of compound **2** in CD_3OD 10

S17. ESIMS spectrum of compound **3** 11

S18. IR spectrum of compound **3** 12

S19. ^{1}H NMR spectrum (400 MHz) of compound **3** in $CDCl_3$ 12

S20. ^{13}C NMR spectrum (100 MHz) of compound **3** in $CDCl_3$ 13

S21. DEPT spectrum of compound **3** in $CDCl_3$ 13

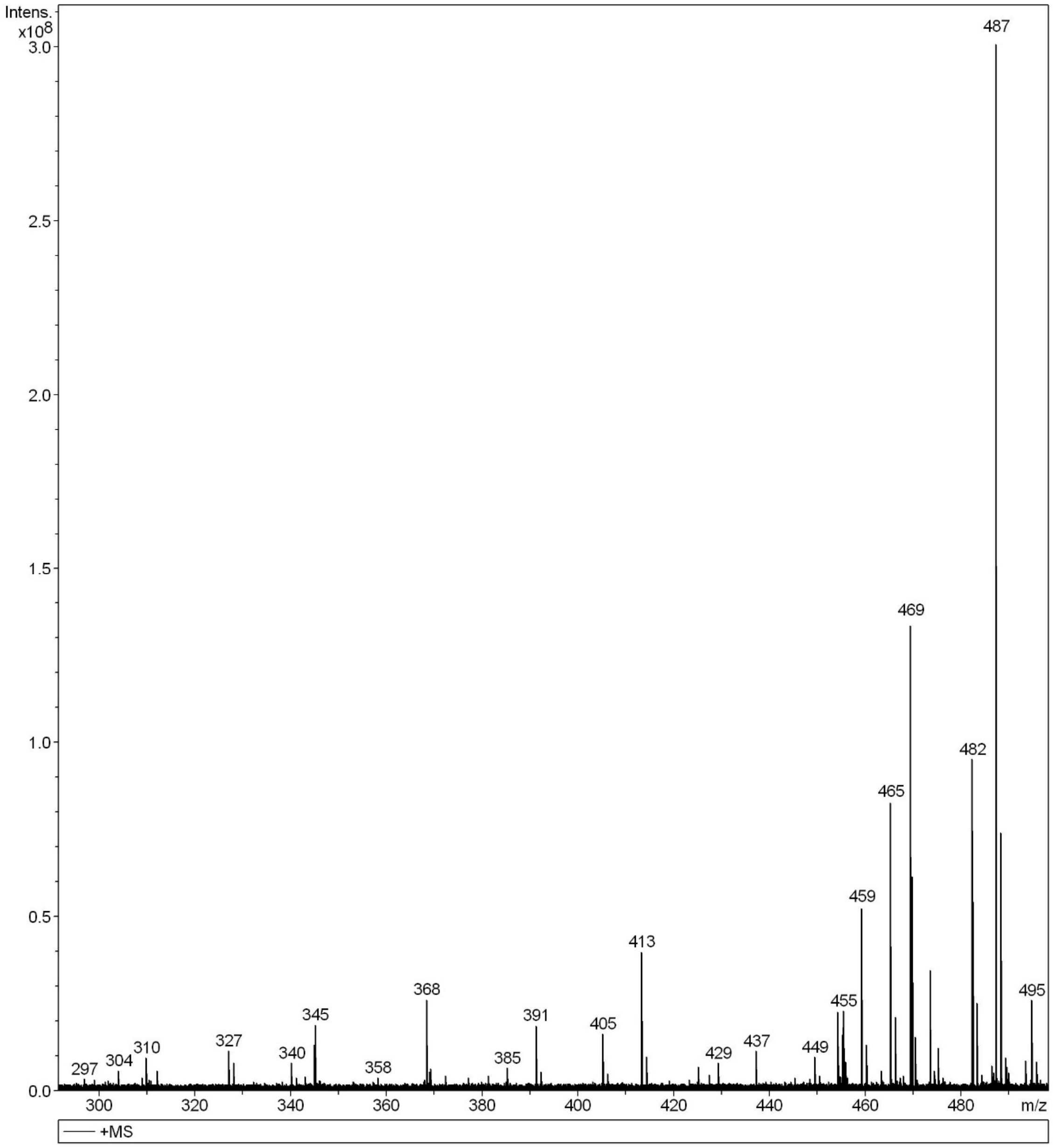

S1. ESIMS spectrum of compound **1**

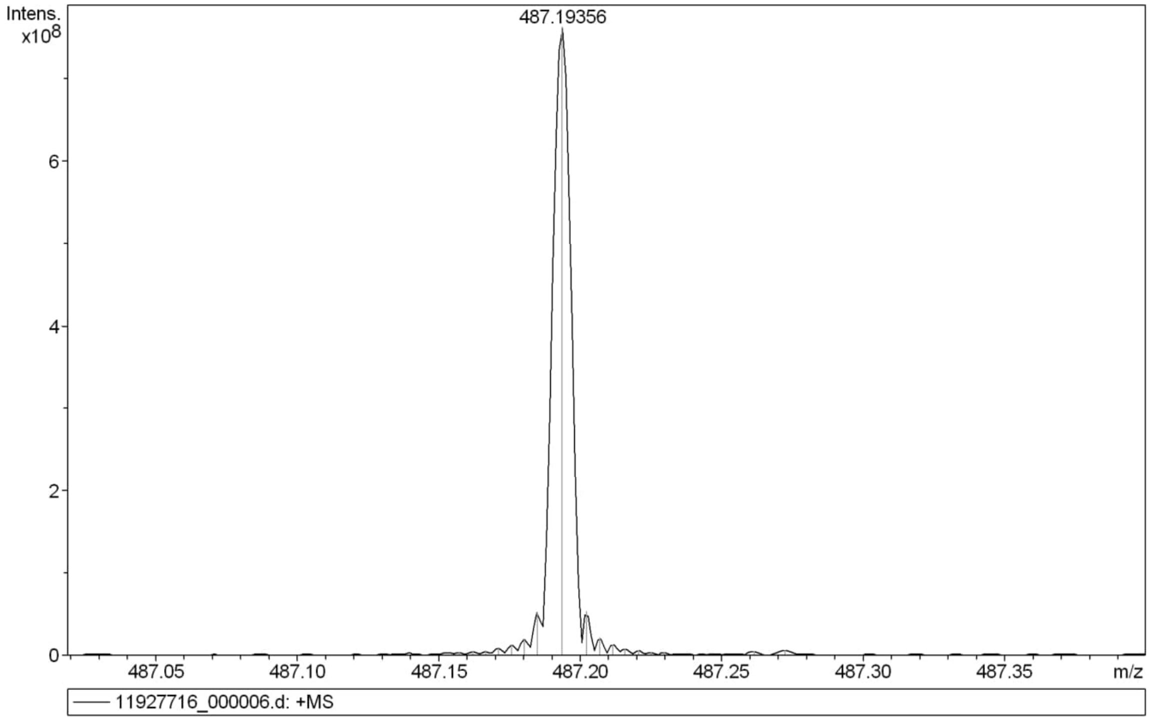

Meas. m/z	#	Formula	Score	m/z	err [mDa]	err [ppm]	mSigma	rdb	e⁻ Conf	N-Rule
487.19356	1	C 24 H 32 Na O 9	100.00	487.19385	0.30	0.61	9.3	8.5	even	ok

S2. HRESIMS spectrum of compound **1**

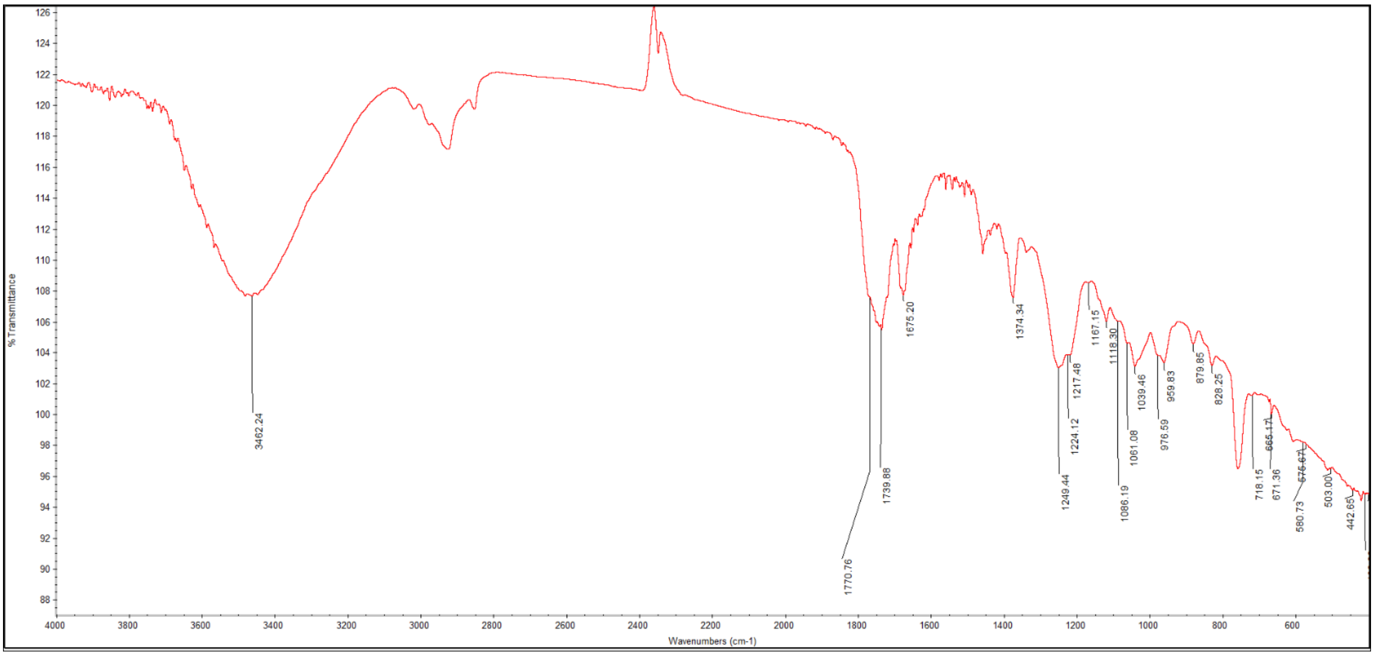

S3. IR spectrum of compound **1**

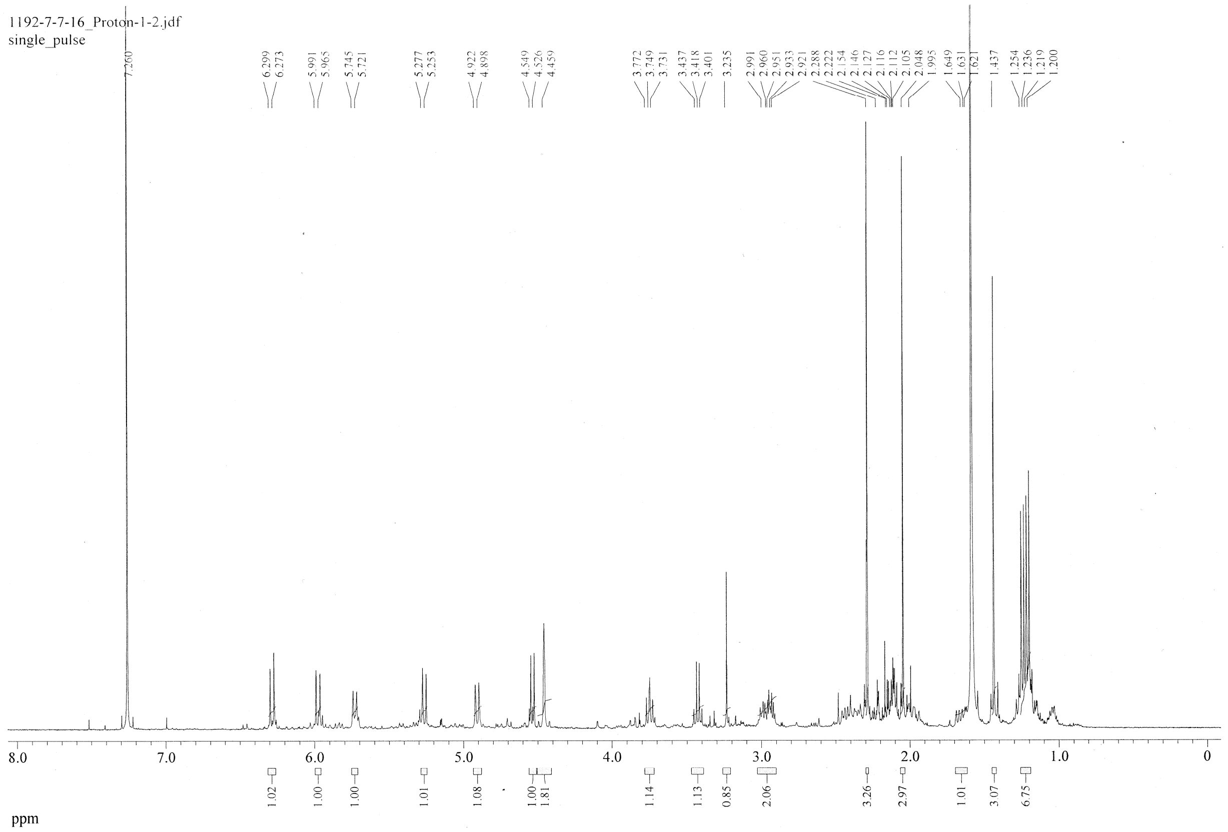

S4. ^{1}H NMR spectrum (400 MHz) of compound **1** in $CDCl_3$

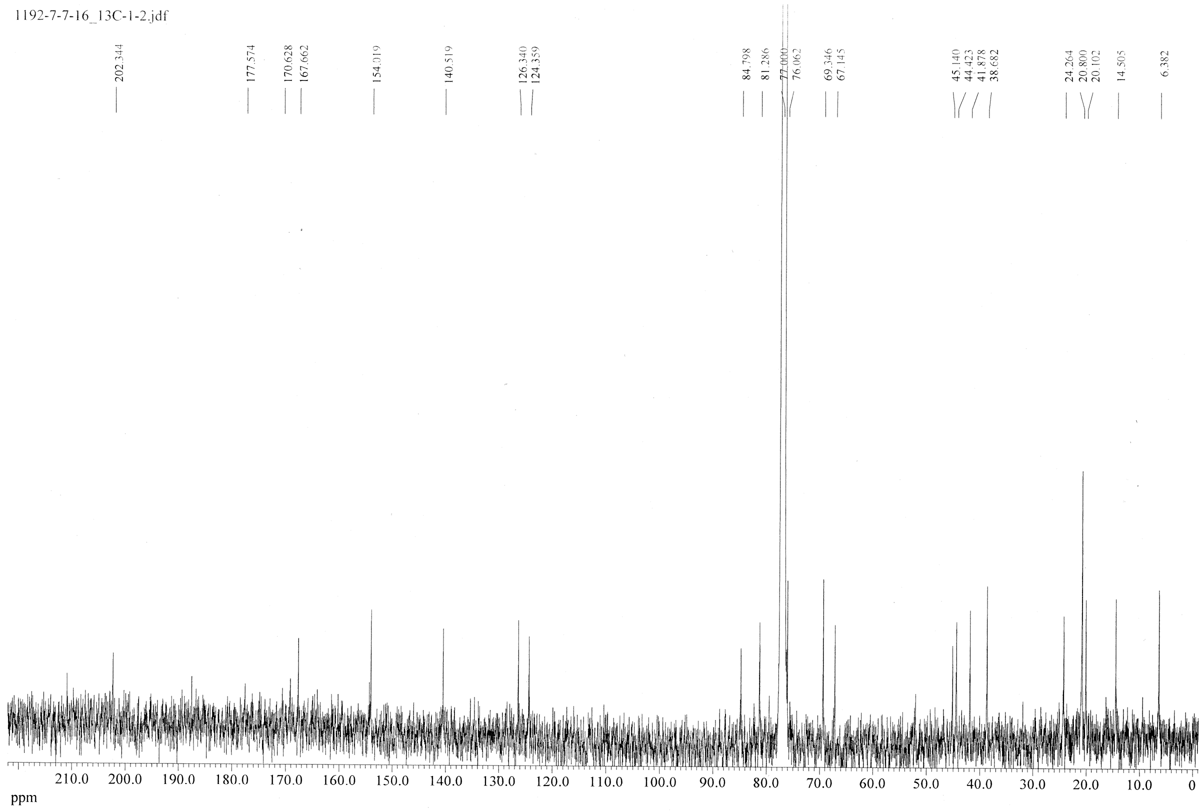

S5. ^{13}C NMR spectrum (100 MHz) of compound **1** in $CDCl_3$

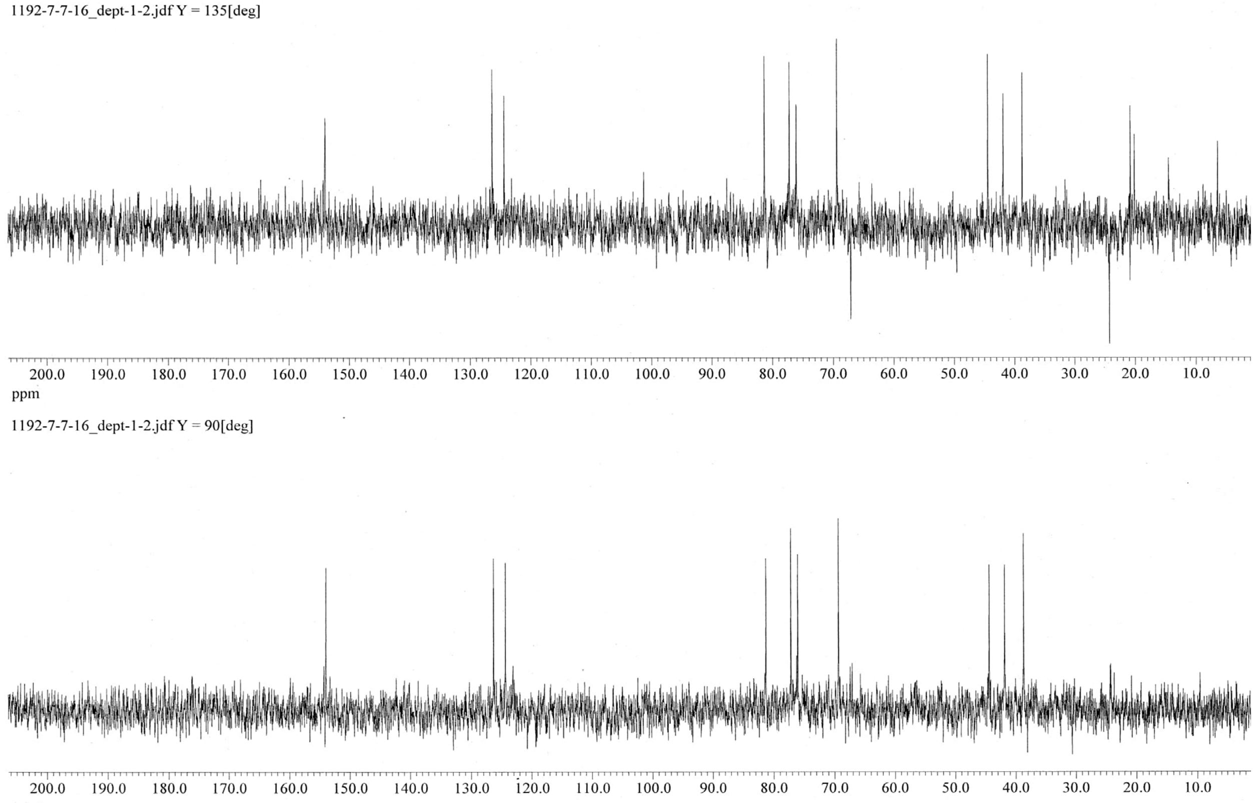

S6. DEPT spectrum of compound **1** in $CDCl_3$

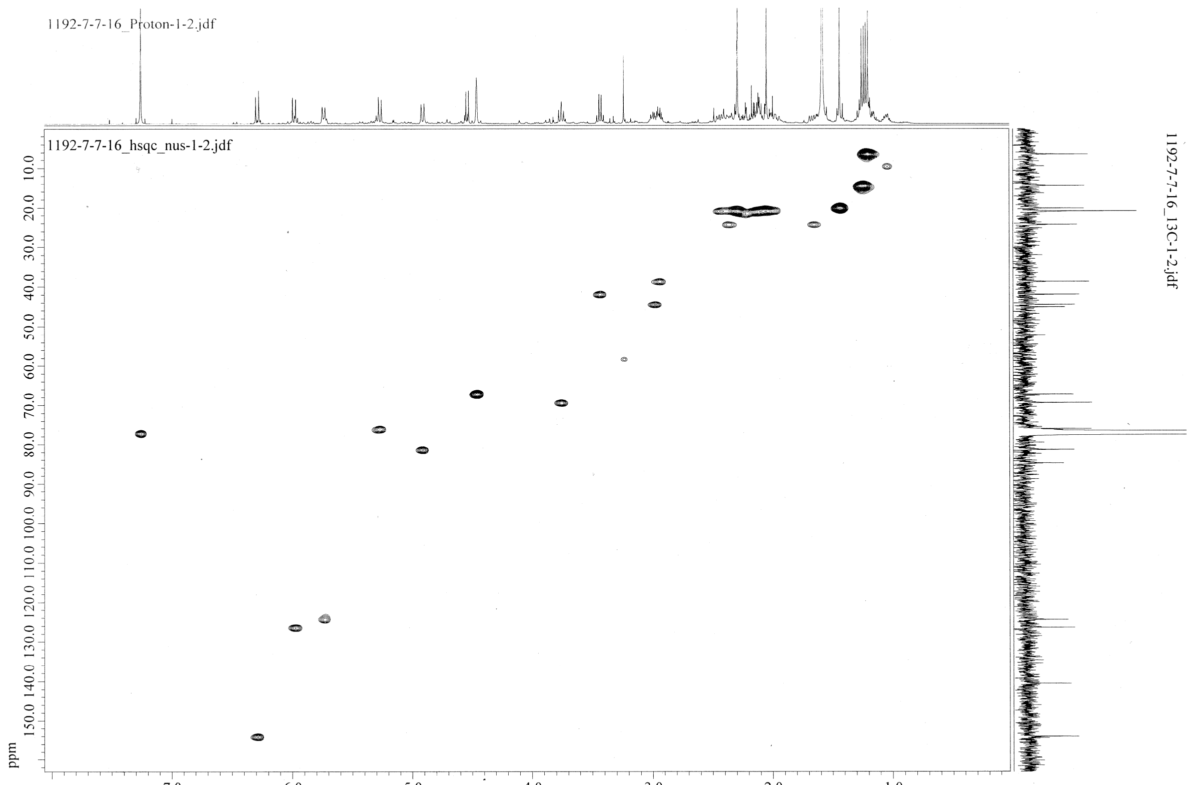

S7. HSQC spectrum of compound **1** in $CDCl_3$

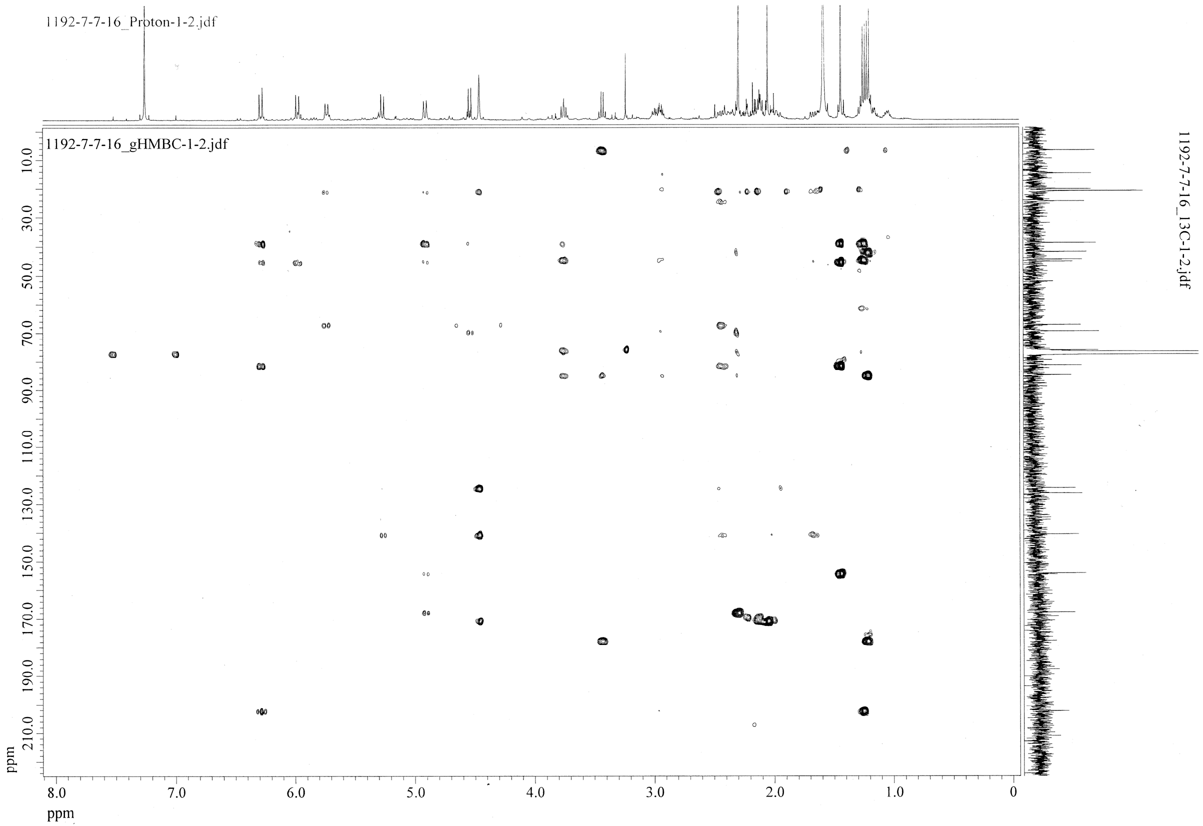

S8. HMBC spectrum of compound **1** in $CDCl_3$

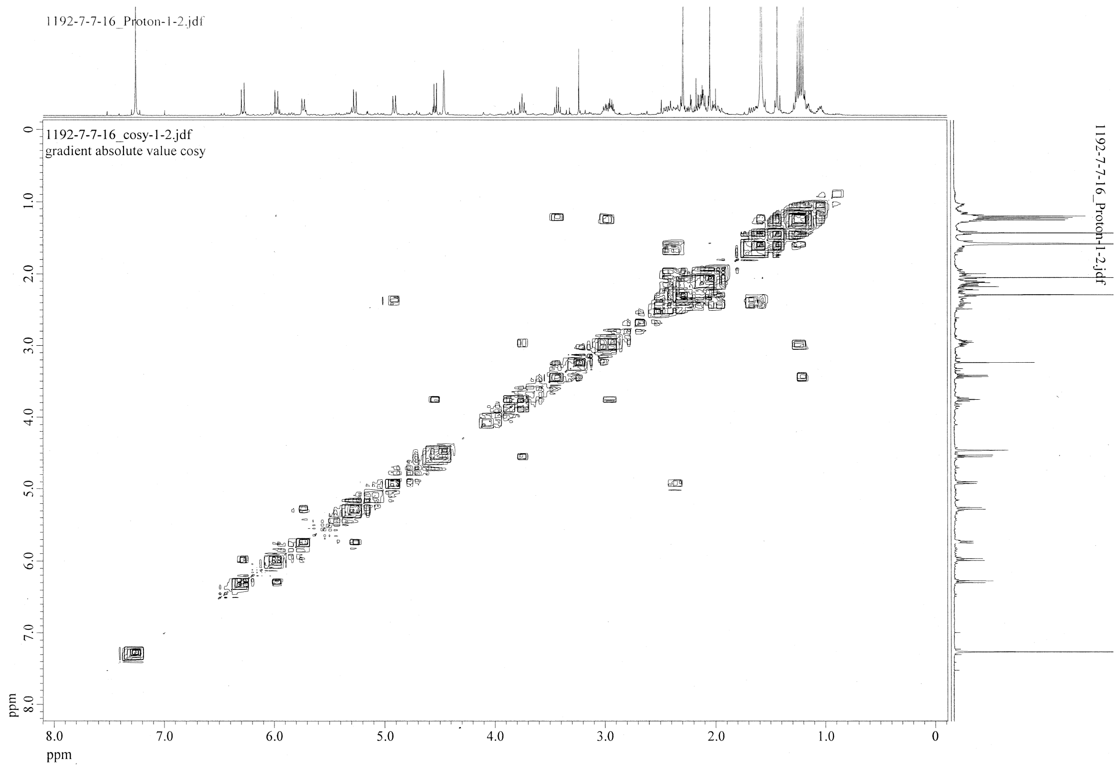

S9. ^1H-^1H COSY spectrum of compound **1** in $CDCl_3$

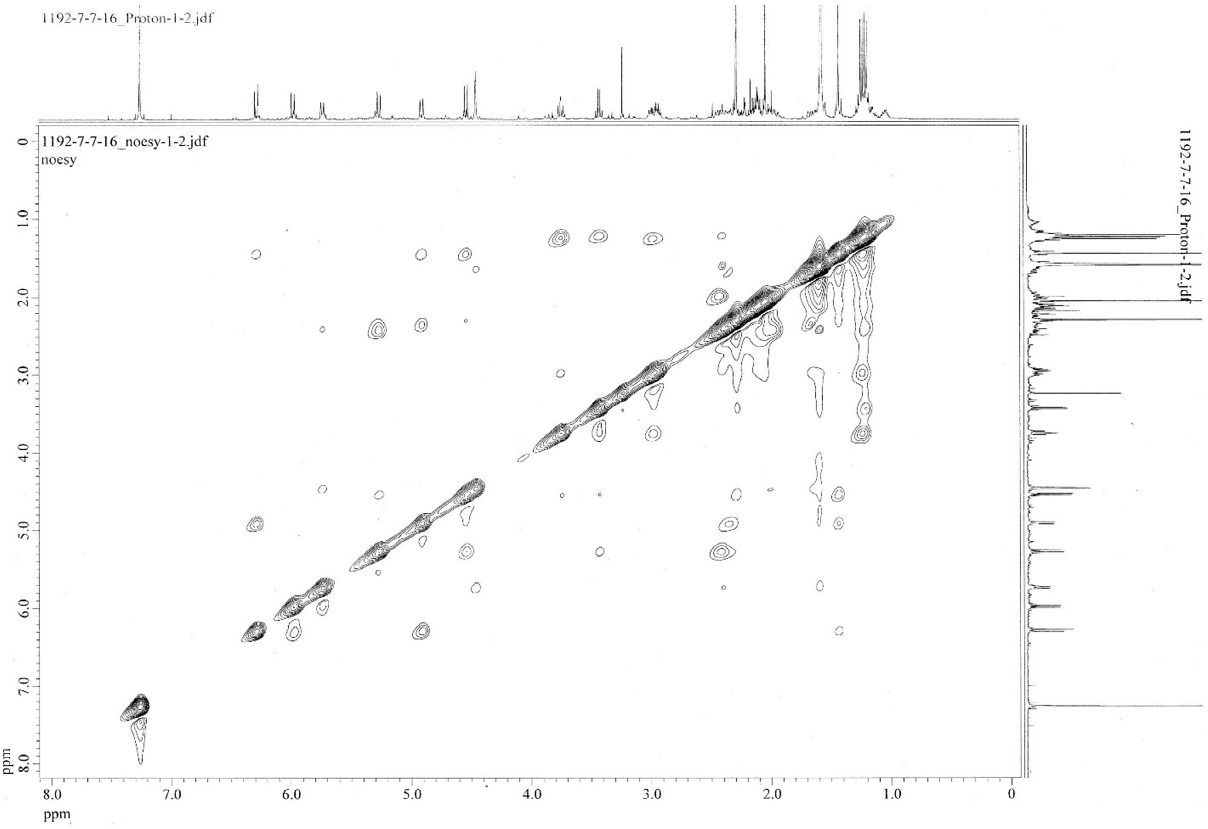

S10. NOESY spectrum of compound **1** in $CDCl_3$

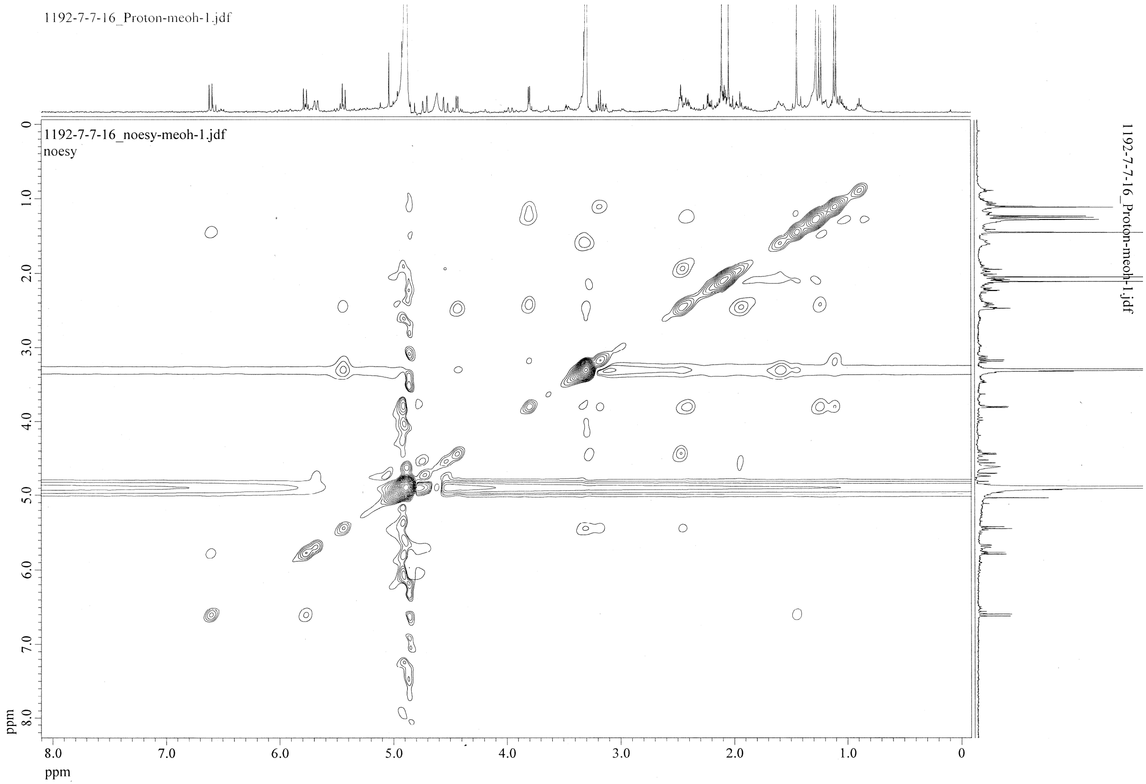

S11. NOESY spectrum of compound **1** in CD_3OD

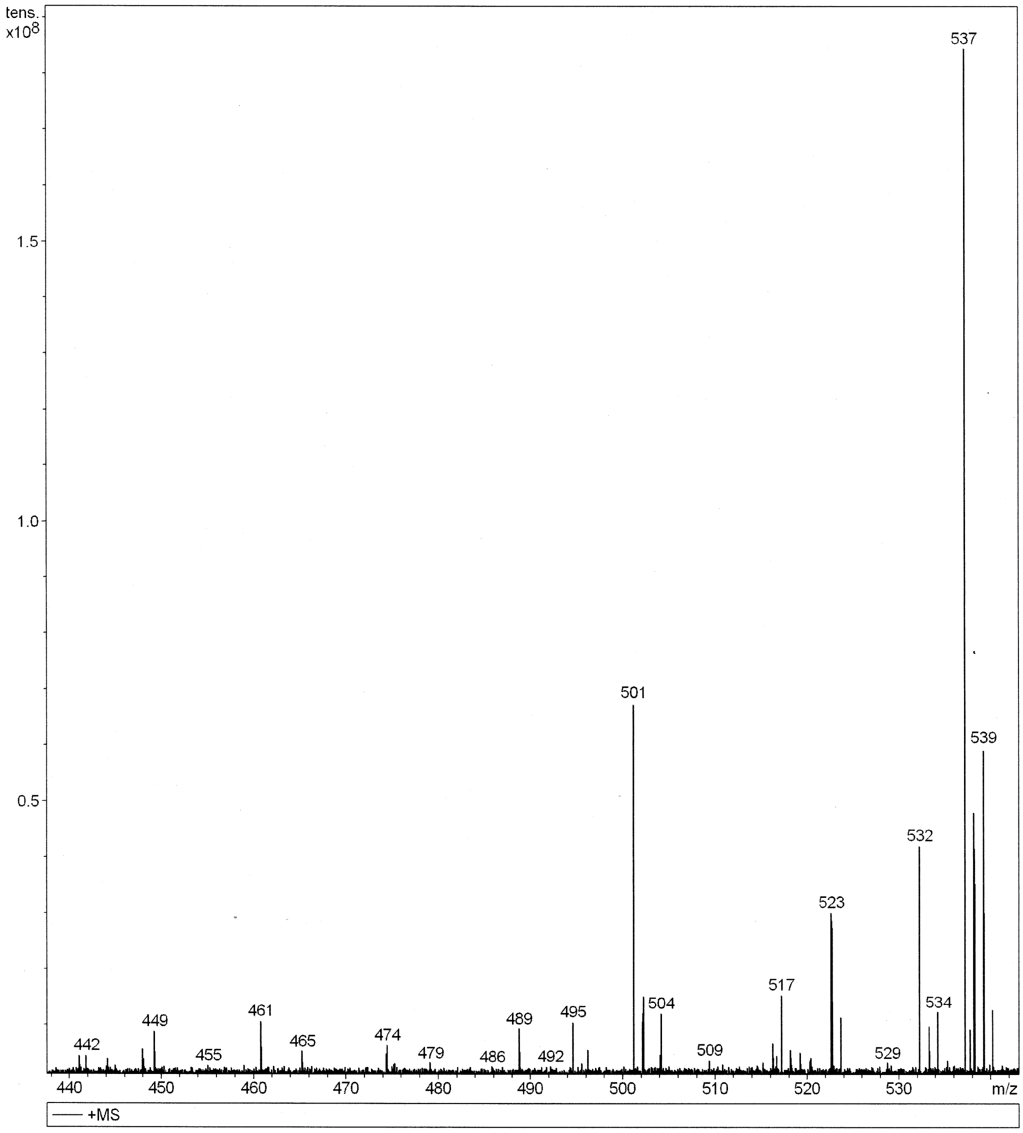

S12. ESIMS spectrum of compound **2**

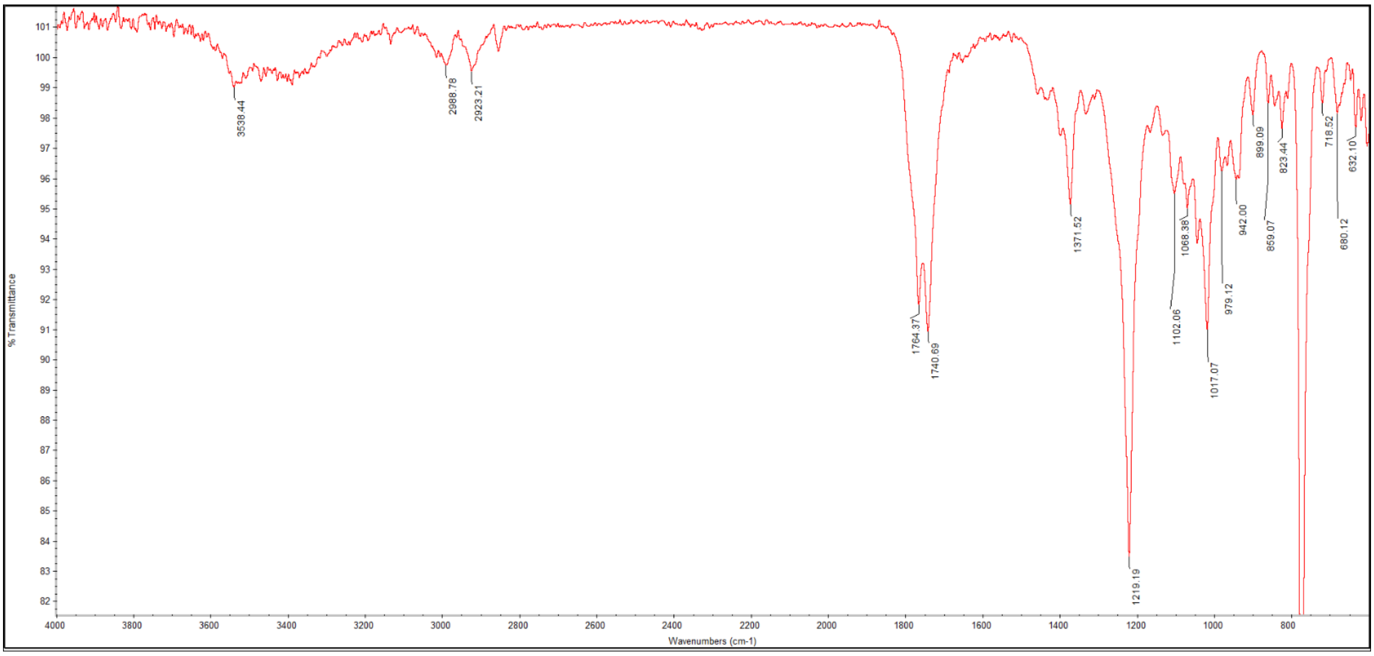

S13. IR spectrum of compound **2**

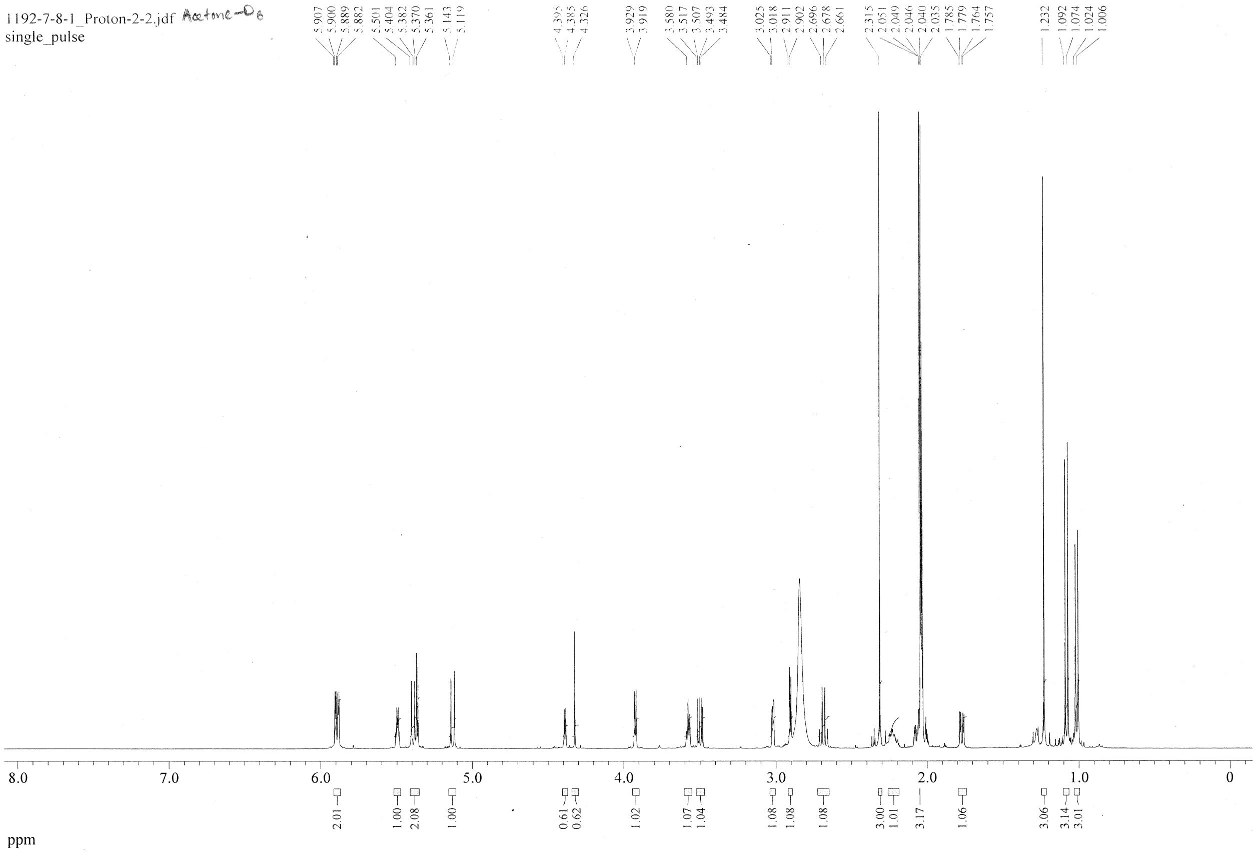

S14. ^{1}H NMR spectrum (400 MHz) of compound **2** in CD_3COCD_3

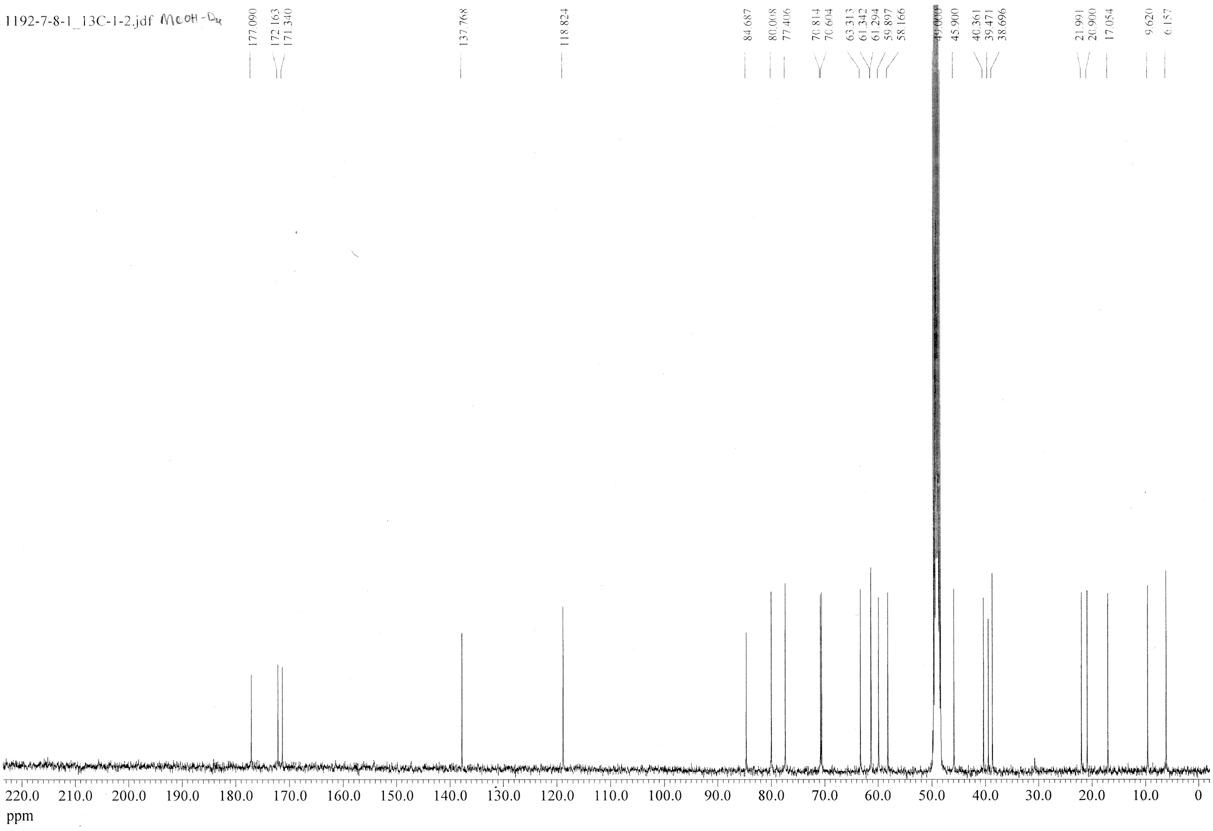

S15. ^{13}C NMR spectrum (100 MHz) of compound **2** in CD_3OD

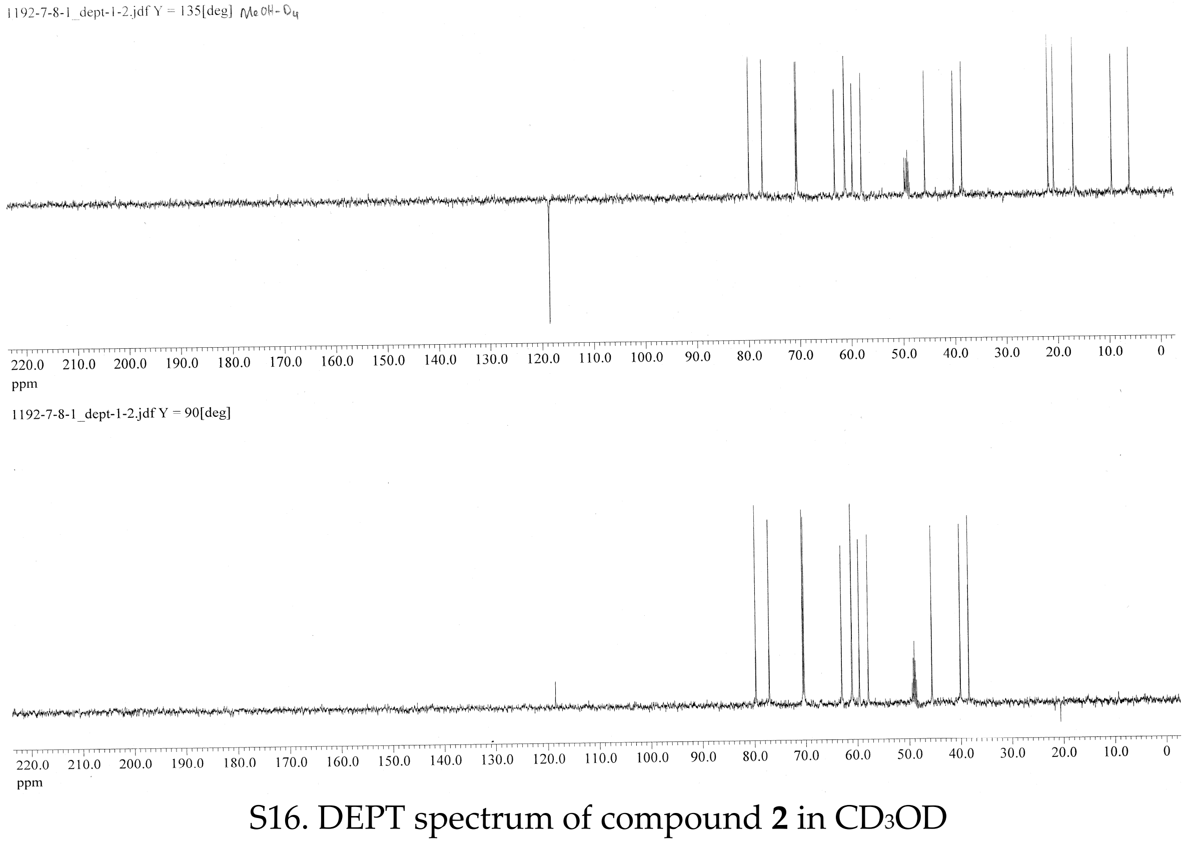

S16. DEPT spectrum of compound **2** in CD_3OD

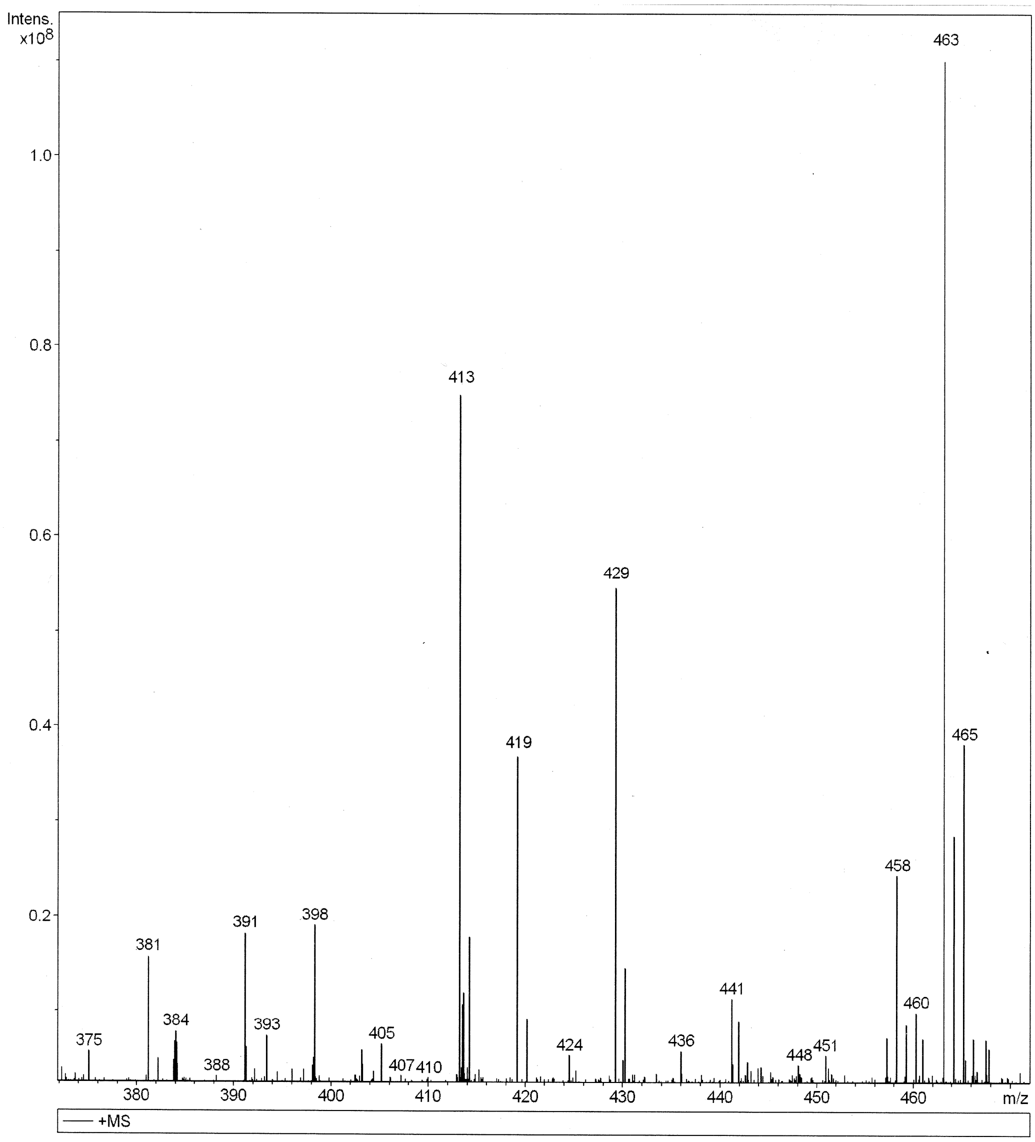

S17. ESIMS spectrum of compound **3**

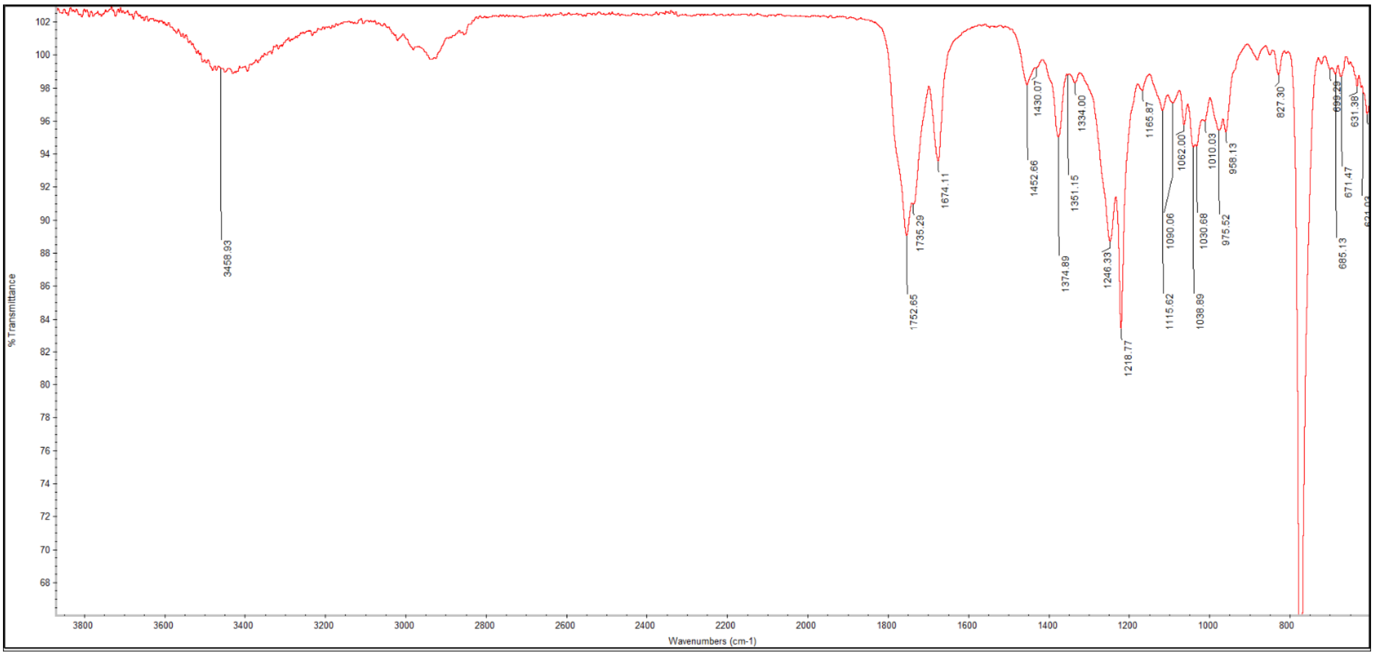

S18. IR spectrum of compound **3**

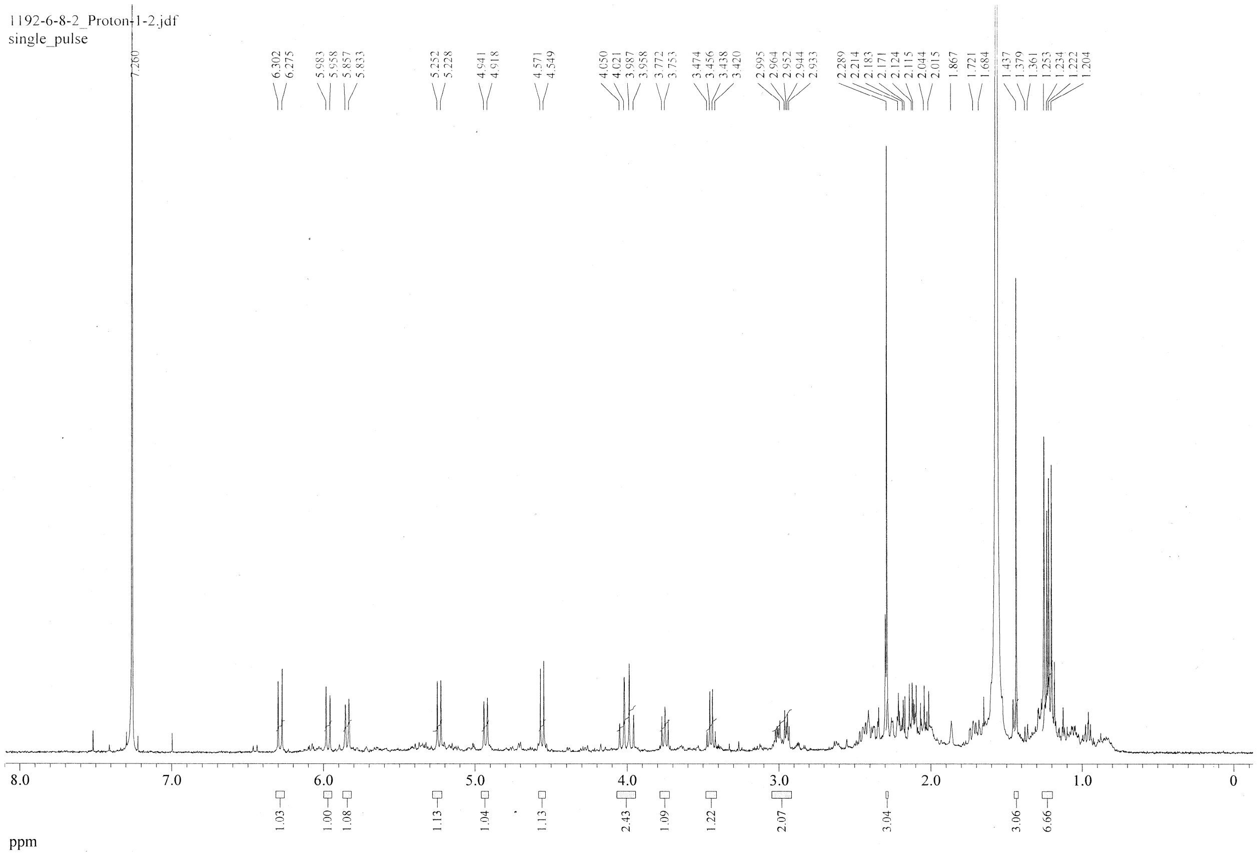

S19. ¹H NMR spectrum (400 MHz) of compound **3** in $CDCl_3$

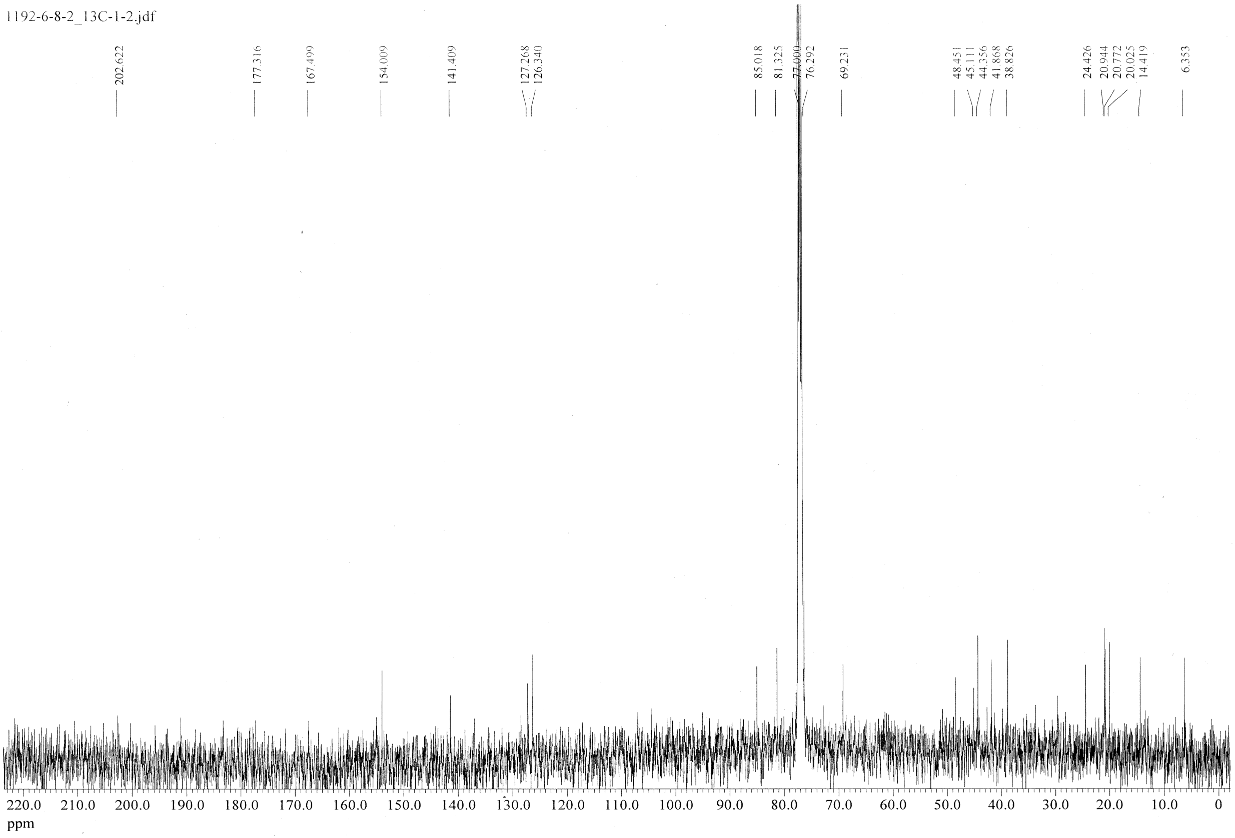

S20. ^{13}C NMR spectrum (100 MHz) of compound **3** in $CDCl_3$

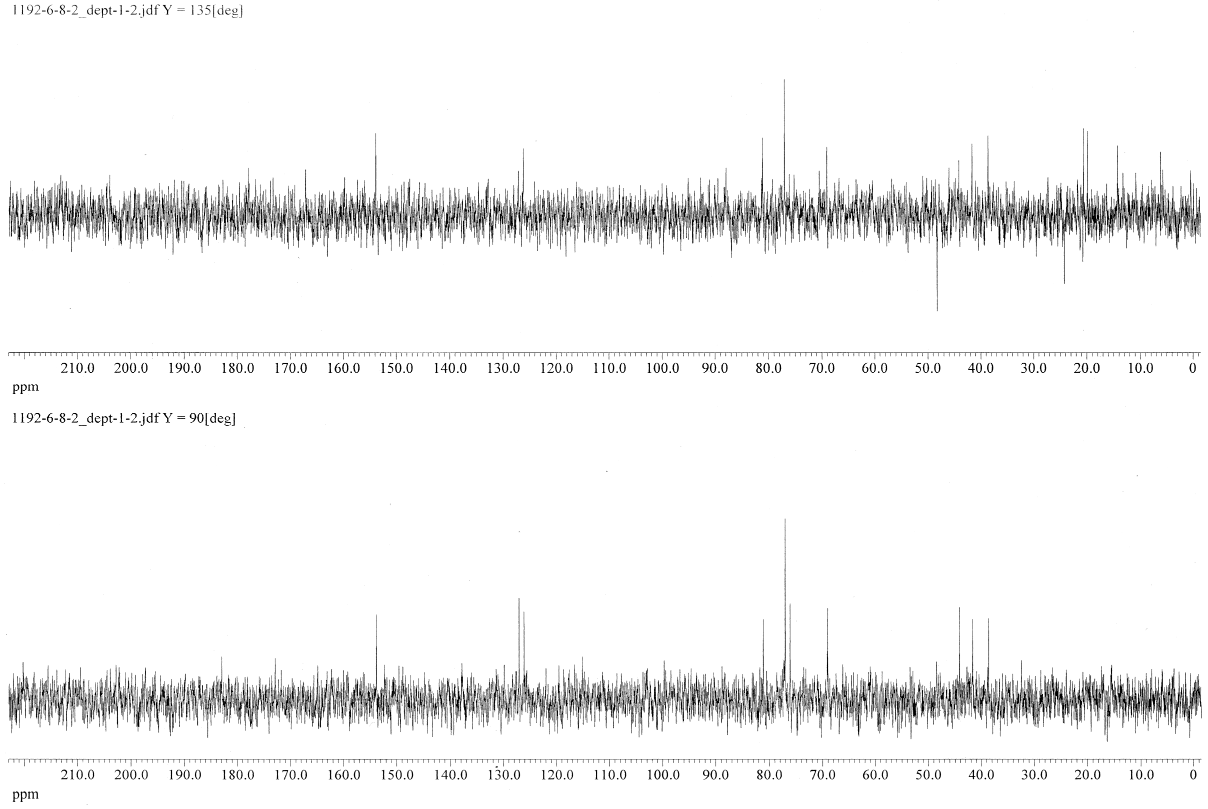

S21. DEPT spectrum of compound **3** in $CDCl_3$
